# Oculomotor impairments in *de novo* Parkinson’s disease

**DOI:** 10.3389/fnagi.2022.985679

**Published:** 2022-11-09

**Authors:** Meng-Xi Zhou, Qin Wang, Yin Lin, Qian Xu, Li Wu, Ya-Jing Chen, Yu-Han Jiang, Qing He, Lei Zhao, You-Rong Dong, Jian-Ren Liu, Wei Chen

**Affiliations:** ^1^Department of Neurology, Shanghai Ninth People’s Hospital, Shanghai Jiao Tong University School of Medicine, Shanghai, China; ^2^Department of Neurology, Zhongshan Hospital Fudan University, Shanghai, China; ^3^Clinical Research Center, Shanghai Jiao Tong University School of Medicine, Shanghai, China

**Keywords:** Parkinson’s disease, essential tremor, ocular movement, videonystagmography, motor symptom

## Abstract

**Objective:**

Reliable electrophysiological indicators are urgently needed in the precise evaluation of Parkinson’s disease (PD). It is still elusive whether oculomotor performance is impaired or has clinical value in early PD. This study aims to explore oculomotor performance in newly diagnosed, drug-naïve PD and its correlation with clinical phenotype.

**Methods:**

Seventy-five patients with *de novo* PD, 75 patients with essential tremor (ET), and 46 gender-and age-matched healthy controls (HCs) were included in this cross-sectional study. All subjects underwent oculomotor test *via* videonystagmography. Visually guided saccade latency, saccadic accuracy and gain in smooth pursuit eye movement (SPEM) at three frequencies of the horizontal axis were compared among the three groups. Patients with PD also received detailed motor and non-motor evaluation by serial scales. The association between key oculomotor parameters and clinical phenotypes were explored in PD patients.

**Results:**

Both *de novo* PD and ET patients showed prolonged saccadic latency and decreased saccadic accuracy relative to HCs. SPEM gain in PD was uniformly reduced at each frequency. SPEM gain at 0.4 Hz was also decreased in ET compared with HCs. However, there was no significant difference of oculomotor parameters between *de novo* PD and ET patients. Furthermore, prolonged saccadic latency was correlated with long disease duration, whereas decreased SPEM gain was associated with severe motor symptoms in *de novo* PD patients.

**Conclusion:**

Ocular movements are impaired in *de novo*, drug naïve PD patients; these changes could be indicators for disease progression in PD.

## Introduction

Parkinson’s disease (PD) is a common neurodegenerative disease characterized by the progressive loss of dopaminergic neurons in the substantia nigra, contributing to serial motor and non-motor symptoms. Diagnostic and prognostic biomarkers are pivotal in the precise evaluation of PD, as this disease is usually detected in the late stage when dopaminergic neurons have degenerated completely (Lotankar et al., 2017). Clinical electrophysiological studies demonstrated that PD patients also had various oculomotor abnormalities (Guo et al., 2018). The investigation of oculomotor system by recording eye movements provides valuable information about the pathophysiology of PD. As ocular movements can be measured non-invasively and precisely using an infrared eye tracker system, oculomotor alterations have gained great interest as a potential electrophysiological biomarker for precise evaluation of early PD.

Accumulating evidence since 1983 showed that PD patients exhibited oculomotor deficits in saccadic and smooth pursuit eye movement (SPEM) systems relative to the healthy controls (HCs; White et al., 1983; Jung and Kim, 2019). However, ocular impairments in PD vary depending on disease stage and current medication is one of the major challenges in interpretation of the results. In previous studies, improvement of saccade and SPEM in PD patients during treatment with levodopa has been observed (Bares et al., 2003; Michell et al., 2006; Marino et al., 2010). It was reported that cholinergic therapies may increase saccadic latency and reduce amplitude or gain (Naicker et al., 2017). As a result, it is difficult to determine whether deficits occur because of underlying PD pathology or the anti-parkinsonian medications. It was reported that PD patients in the ‘off’ medication state may exhibit less fixation stability, longer saccadic latency and decreased SPEM gain compared to normal subjects (MacAskill et al., 2002; Marino et al., 2010; Shaikh and Ghasia, 2019). However, most of these studies were observed in the mild-to-moderate stage of the disease. Few studies have explored this issue with patients in the very early stage of the disease, especially *de novo*, drug-naïve patients (Bares et al., 2003; Marino et al., 2010; Linder et al., 2012).

Essential tremor (ET) has been regarded as a monosymptomatic entity characterized by action tremor involving mainly hands and forearms (Haubenberger and Hallett, 2018). The differential diagnosis of ET and PD can be very challenging, especially in the early course of the disease. Recently, the presence of eye movement disturbances including saccadic and SPEM system were also described in ET patients and cerebellar dysfunction may be the cause (Wójcik-Pędziwiatr et al., 2016). Patterns of abnormal ocular movements were supposed to provide a key for differential diagnosis between these two diseases.

The current study aims to (1) establish whether ocular impairment is present in early PD before the introduction of any medication, relative to HCs and ET patients; (2) analyze the association between oculomotor performance and clinical features in PD.

## Materials and methods

### Subjects

We conducted an observational cross-sectional study from Jan 2017 to Dec 2021. This exploratory study included 75 *de novo* patients with PD, 75 patients with ET and 46 age-and gender - matched HCs. Patients were recruited from the Department of Neurology, Shanghai Ninth People’s Hospital, Shanghai Jiao Tong University School of Medicine. Patients with PD were included if they met the Movement Disorder Society criteria (Postuma et al., 2015). None of the patients was under dopaminergic medication or had undergone functional neurosurgery for PD. The diagnosis of ET was made according to the consensus criteria proposed by the Tremor Investigation Group (Deuschl et al., 1998). Overall neurological examinations were conducted by two neurologists and eye movements were visually checked. Individuals with restriction of the eye mobility, red or green color blindness, other chronic or acute brain diseases were excluded. 46 HCs were recruited from the local community. Written informed consents were obtained from all participants. The study was approved by the Ethics Committee of Shanghai Ninth People’s Hospital, Shanghai Jiao Tong University School of Medicine.

### Clinical and neuropsychological assessments

For PD patients, motor severity was measured with the modified Hoehn and Yahr (H&Y) stage (1967), Unified Parkinson’s Disease Rating Scale part III (UPDRS-III; Richards et al., 1994) and the Freezing of Gait questionnaire (Giladi et al., 2000). Motor subtype (tremor-dominant, akinetic-rigid, mixed) was further defined according to the report from Kang et al. (2005). For ET patients, tremor severity was measured by the Tremor Research Group Essential Tremor Rating Scale (TETRAS; Elble et al., 2012). REM Behavior Disorder Screening Questionnaire (RBDSQ) was used as a screening tool of clinical possible RBD (Nomura et al., 2011).The total burden of non-motor symptoms was measured with Non-Motor Symptoms Questionnaire (NMSQuest; Richards et al., 1994). Olfactory function was assessed by the 16-item odor identification test from Sniffin’ Sticks (SS-16; Burghart Messtechnik, Wedel, Germany) as our previous report (Chen et al., 2012). The severity of depressive symptom was assessed using the 17-item Hamilton Rating Scale for Depression (HAMD-17; Hamilton, 1960). Total cognitive function was assessed by Chinese version of Mini-Mental State Examination (MMSE; Katzman et al., 1988) and Montreal Cognitive Assessment Basic (MoCA-BC; Xu et al., 2021).

### Videonystagmography evaluation

All the subjects underwent oculomotor test by a Visual Eyes 4 channel VNG (Micromedical Technologies, USA), which acquired binocular movement samples at 120 Hz. The video-based eye tracker used the center of the pupil to measure the coordinate of the gaze position. Subjects were seated at a distance of 100 cm in front of the screen and remained seated in darkness for 2 min before testing. Participants were required to keep their heads stationary, while moving their eyes according to instructions on screen.

In the saccade task, subjects were required to fixate a small white target on the central spot (0°). The target was stepped 10° or 20° at intervals greater than 2 s, in pseudorandom directions (right or left). The subject was instructed to visually track the target light as rapidly as possible. Each participant was tested 15 times on each side, across a total of 4 amplitudes (10°, 20°, 30°, or 40°). Saccadic latency was defined as the time between the appearance of the target and the start of the main saccade; whereas saccadic accuracy was regarded as saccadic amplitude/target amplitude×100%. In the smooth pursuit task, the subjects were required to pursue the target when the target started to move along the horizontal axis. Horizontal SPEM was conducted over 4 cycles while tracking targets at three frequencies (0.1, 0.2, and 0.4 Hz). SPEM gain was defined as the mean velocity of eye movement/velocity of the target. The oculomotor parameters: mean saccadic latency, mean saccadic accuracy, and gain of SPEM at three different frequencies were automatically recorded and calculated by the analysis system of the machine. In addition, we also manually checked the results. Misdirected visually guided saccade (VGS) and ocular movements that involving blinking or measurement errors were excluded from analyses.

### Statistical analysis

All analyses were performed with SPSS (version 23.0 for Windows), and figures were generated with GraphPad Prism (version 9.0 for Windows). Continuous variables are expressed as the means ± SD or medians (interquartile ranges (IQR, 25th-75th)). Categorical variables are expressed as frequencies and percentages. To compare categorical data among groups, we applied the chi-square test or Fisher’s exact test. Comparisons of means between the two groups were performed using the independent *t* test or non-parametric Kruskal–Wallis test, depending on whether the data were normally distributed or not. We analyzed continuous variables among HCs, ET and PD by one-way analysis of variance (ANOVA). The least significant difference (LSD) method was adopted for *post hoc* analysis. Linear regression analysis by backward was used to determine the independent associated factors of key oculomotor dynamics in *de novo* PD patients. The linear regression variables included age, sex, UPDRS III, MoCA-BC and those with significant difference in univariate analysis (*value of p* <0.1). *β* value and 95% confidence intervals (CIs) were reported accordingly. Diagnostic accuracy was evaluated by receiver operating characteristic (ROC) curve analysis. Area under the curve (AUC), sensitivity and specificity were calculated accordingly for each oculomotor parameter. In all analyses, a two tailed *value of p* <0.05 was considered statistically significant.

## Results

### Demographic and clinical features of *de novo* PD patients and ET patients

The general characteristics and clinical features of patients with *de novo* PD and ET were shown in Table 1. Age, gender, education level and MMSE scores were similar among the three groups, whereas ET patients had an earlier onset age (*p* < 0.001) and longer disease duration (*p* < 0.001). In addition, PD had more non-motor symptom burden as shown with higher scores of NMSQuest (*p* = 0.001), RBDSQ (*p* = 0.001), HAMD-17 (*p* = 0.013), and lower score of SS-16 (*p* < 0.001).

**Table 1:** Demographic and clinical features of *de novo* PD and ET patients.

Items	HC	ET	PD	*p*-value
Number, *n*	46	75	75	
Age, years	62.6 ± 7.9	62.9 ± 12.9	64.5 ± 8.1	0.324
Male, *n* (%)	29 (63.0)	42 (56.0)	40 (53.3)	0.572
Disease duration, months	NA	60 (24–120)	12 (6–24)	^a^ **<0.001** ^***^
Age at onset, years	NA	53.5 ± 15.4	62.9 ± 8.4	^a^ **<0.001** ^***^
Education, years	9 (9–12)	9 (9–12)	9 (9–12)	0.881
Motor features in PD				
Hoehn and Yahr stage ≥ 2 (%)	NA	NA	38 (50.7)	NA
UPDRS-III	NA	NA	19 (13–29)	NA
Motor subtype (%)				
Tremor-dominant type	NA	NA	23 (30.7)	NA
Akinetic-rigid type	NA	NA	44 (58.7)	NA
Mixed type	NA	NA	8 (10.7)	NA
FOG-Q	NA	NA	1 (0–4)	NA
Non-motor features				
NMSQuest	NA	4.7 ± 4.8	6.9 ± 4.3	^a^ **0.001** ^**^
SS-16	NA	11 (10–13)	8 (6–10)	^a^ **<0.001** ^***^
RBDSQ	NA	1 (1–3.25)	3 (1–6)	^a^ **0.001** ^**^
HAMA-17	NA	2.5 (0–5)	4 (2–9)	^a^ **0.013** ^*^
MMSE	27.2 ± 1.5	27.7 ± 1.9	27.6 ± 2.3	0.767
MoCA-BC	NA	23 ± 3.4	22.7 ± 4.6	^a^0.738
Clinical evaluation in ET				
TETRAS	NA	15.13 ± 5.4	NA	NA
TETRAS-ADL	NA	11.55 ± 7.4	NA	NA

a Compared between ET and PD.

HC, healthy control; ET, essential tremor; PD, Parkinson’s disease; UPDRS, Unified Parkinson’s Disease Rating Scale; FOG-Q, Freezing of Gait-Questionnaire; NMSQuest, Non-motor Symptoms Questionnaire; SS-16, 16-item odor identification test from Sniffin’ Sticks; RBDSQ, REM Behavior Disorder Screening Questionnaire; HAMD-17, 17-item Hamilton Rating Scale for Depression; SCOPA-AUT, The Scale for Outcomes in PD autonomic dysfunction; MMSE, Mini Mental State Examination; MoCA-BC, Montreal Cognitive Assessment Basic; TETRAS, Tremor Research Group Essential Tremor Rating Scale; TETRAS-ADL, Tremor Research Group Essential Tremor Rating Scale-Activities of daily living; NA, not applicable.

Values were presented as mean ± SD or median (IQR) or number (proportion).

Statistically significant *p* values were shown in bold. ^*^p < 0.05; ^**^p < 0.01; ^***^p < 0.001.

### Oculomotor performances in ET patients and *de novo* PD patients

**Figure 1:**
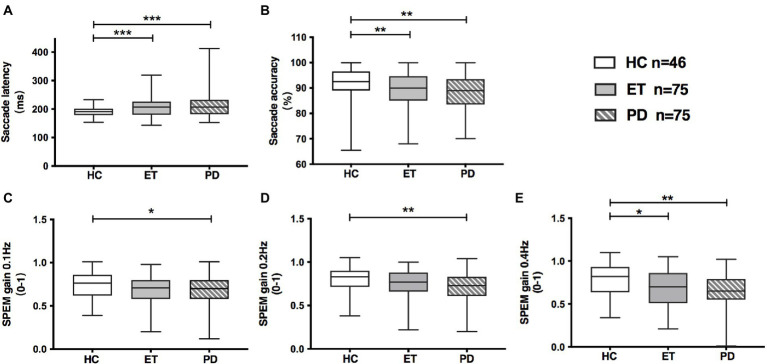
Oculomotor parameters in HC, ET and *de novo* PD patients. **(A)** Saccadic latency was increased both in ET and PD group, compared with HC group; **(B)** Saccadic accuracy was decreased both in ET and PD group, compared with HC group; **(C,D)** PD group had decreased gain of smooth pursuit eye movement at 0.1 Hz and 0.2 Hz, while ET group had marginally decreased gain of smooth pursuit eye movement at 0.1 Hz and 0.2 Hz, relative to HC group; **(E)** ET and PD patients had decreased gain of smooth pursuit eye movement at 0.4 Hz, relative to HCs. Box and whiskers (minimum and maximum) were presented for each group. Middle bars inside boxes represent median, while the edges of boxes indicate first and third quartiles. HC, healthy control; ET, essential tremor; PD, Parkinson’s disease; SPEM, smooth pursuit eye movement; *p < 0.05; ^**^p < 0.01; ^***^p < 0.001.

Both ET and PD group showed prolonged saccadic latency (ET: 209 ms, PD: 210 ms, *p* < 0.001) and decreased saccadic accuracy (ET: 89.2%, PD: 88.4%, *p* < 0.05) relative to HC group (Table 2; Figures 1A,B). Meanwhile, SPEM gain at 0.1 Hz (*p* = 0.026), 0.2 Hz (*p* = 0.008) and 0.4 Hz (*p* = 0.004) was significantly decreased in PD compared with HC group (Table 2; Figures 1C–E). ET patients also had decreased SPEM gain at 0.4 Hz (*p* = 0.014, Table 2; Figure 1E) in comparison with HCs. SPEM gain at 0.4 Hz was not associated with tremor severity in ET as revealed by TETRAS score (*r* = −0.230, *p* = 0.103). There was a trend that ET group had low SPEM gain at 0.1 Hz (*p* = 0.062) and 0.2 Hz (*p* = 0.063), but without statistical significance. No obvious difference was observed in saccadic latency, saccadic accuracy and SPEM gain between the ET and PD group.

**Table 2:** Oculomotor characteristics in HCs, ET, and *de novo* PD patients.

Items	HC	ET	PD	*p*-value		
	Group A	Group B	Group C	A vs. B	A vs. C	B vs. C
Number, *n*	46	75	75			
Saccadic latency, ms	191.3 ± 18.9	209.1 ± 34.9	210.4 ± 41.3	**0.007** ^**^	**0.004** ^**^	0.820
Saccadic accuracy, 0–100%	92.2 ± 6.1	89.2 ± 6.7	88.4 ± 6.8	**0.019** ^*^	**0.003** ^**^	0.456
SPEM gain 0.1 Hz, 0–1	0.74 ± 0.14	0.69 ± 0.17	0.68 ± 0.15	0.055	**0.034** ^*^	0.812
SPEM gain 0.2 Hz, 0–1	0.79 ± 0.16	0.74 ± 0.17	0.72 ± 0.16	0.06	**0.009** ^**^	0.406
SPEM gain 0.4 Hz, 0–1	0.78 ± 0.19	0.69 ± 0.19	0.67 ± 0.19	**0.014** ^*^	**0.004** ^**^	0.614

HC, healthy control; ET, essential tremor; PD, Parkinson’s disease; SPEM, smooth pursuit eye movements.

Values were presented as mean ± SD.

Statistically significant *p* values were shown in bold.

^*^p < 0.05; ^**^p < 0.01.

We performed the ROC analyses of eye movement parameters as independent factors for detecting PD from HCs. However, the AUC of each parameter was lower than 0.7 (Supplementary Table 1). Combining the saccadic latency, saccadic accuracy and the most significant SPEM gain (0.4 Hz) revealed that the model could significantly distinguish PD from HCs with an 80.4% sensitivity and a 73.3% specificity (AUC = 0.78, *p* < 0.001). Only three eye movement parameters were significant in detecting ET from HCs. The combination of the above three parameters could differentiate ET from HCs with a high sensitivity of 84.8%, but a low specificity of 58.1% (AUC = 0.719, *p* < 0.001; Supplementary Table 2). However, oculomotor performance cannot provide additional benefit to distinguish PD from ET.

### Independent associated factors with key oculomotor dynamics in *de novo* PD patients

The independent factors associated with oculomotor dynamics in *de novo* PD were further investigated by linear regression (Tables 3, 4). We found that prolonged saccadic latency was associated with old age (*β* = 1.296, 95% CI: 0.154 to 2.439, *p* = 0.027) and long disease duration (*β* = 0.378, 95% CI: 0.054 to 0.703, *p* = 0.023) by univariate linear regression analysis. After adjusting confounding factors such as sex, the score of UPDRS III and MoCA-BC, only disease duration was an independent factor of prolonged saccadic latency (*β* = 0.334, 95% CI: 0.014 to 0.654, *p* = 0.041; Table 3).

**Table 3:** Independent associated factors of saccadic latency in *de novo* PD patients.

Items	Univariate regression		Multivariate regression	
	*β* (95%CI)	*p*-value	*β* (95%CI)	*p*-value
Age	1.296 (0.154, 2.439)	**0.027** ^*^	1.027 (−0.1, 2.153)	0.073
Sex	−8.511 (−27.578,10.557)	0.377	−7.395 (−25.578,10.788)	0.420
Disease duration	0.378 (0.054, 0.703)	**0.023** ^*^	0.357 (0.039, 0.675)	**0.028** ^*^
UPDRS III	0.607 (−0.131,1.344)	0.105	0.604 (−0.12,1.327)	0.101
MoCA-BC	0.275 (−1.818,2.368)	0.794	0.86 (−1.153,2.873)	0.397

PD, Parkinson’s disease; CI, confidential interval; UPDRS, Unified Parkinson’s Disease Rating Scale. MoCA-BC, Montreal Cognitive Assessment Basic.

Statistically significant *p* values were shown in bold.

^*^p < 0.05.

**Table 4:** Independent associated factors of smooth pursuit gain in *de novo* PD patients.

Items	0.1 Hz				0.2 Hz				0.4 Hz			
	Univariate regression		Multivariate regression		Univariate regression		Multivariate regression		Univariate regression		Multivariate regression	
	*β* (95%CI)	*p*-value	*β* (95%CI)	*p*-value	*β* (95%CI)	*p*-value	*β* (95%CI)	*p*-value	*β* (95%CI)	*p*-value	*β* (95%CI)	*p*-value
Age	−0.002 (−0.007, 0.002)	0.295	−0.001 (−0.006, 0.003)	0.540	−0.002 (−0.007, 0.002)	0.313	−0.001 (−0.006, 0.004)	0.580	−0.003 (−0.008, 0.003)	0.335	−0.002 (−0.008, 0.004)	0.483
Sex	0.031 (−0.039,0.101)	0.383	0.027 (−0.046,0.1)	0.462	0.023 (−0.052,0.098)	0.547	0.012 (−0.068, 0.093)	0.760	0.035 (−0.053,0.122)	0.432	0.024 (−0.066,0.114)	0.598
UPDRS III	−0.004 (−0.007,-0.002)	**0.001** ^**^	−0.004 (−0.008,-0.001)	**0.036** ^*^	−0.003 (−0.006,-0.0004)	**0.023** ^*^	−0.002 (−0.006,0.002)	0.309	−0.005 (−0.008,-0.002)	**0.002** ^**^	−0.006 (−0.011, −0.001)	**0.012** ^**^
FOG-Q	−0.007 (−0.015, 0.001)	0.080	0.003 (−0.008, 0.013)	0.643	−0.008 (−0.016, 0.0002)	0.058	−0.002 (−0.014, 0.01)	0.789	−0.008 (−0.018, 0.002)	0.097	0.005 (−0.009, 0.018)	0.470
MoCA-BC	0.004 (−0.003, 0.012)	0.265	0.02 (−0.006, 0.01)	0.664	0.003 (−0.005, 0.011)	0.451	0.001 (−0.007, 0.01)	0.735	−0.003 (−0.012, 0.007)	0.570	−0.007 (−0.016, 0.003)	0.183
NMS-quest	−0.008 (−0.016, 0.001)	0.066	−0.001 (−0.012, 0.01)	0.873	−0.007 (−0.016, 0.002)	0.124	0.001 (−0.013, 0.012)	0.939	−0.008 (−0.018, 0.002)	0.112	−0.003 (−0.017, 0.011)	0.696
HAMD17	−0.006 (−0.012, 0.001)	0.075	−0.002 (−0.011, 0.007)	0.655	−0.005 (−0.012, 0.002)	0.181	−0.003 (−0.012, 0.007)	0.603	−0.006 (−0.014, 0.003)	0.179	0.001 (−0.01, 0.012)	0.874

PD, Parkinson’s disease; CI, confidential interval; UPDRS, Unified Parkinson’s Disease Rating Scale; FOG-Q, Freezing of Gait-Questionnaire; MoCA-BC, Montreal Cognitive Assessment Basic; NMS-Quest, Non-motor Symptoms Questionnaire; HAMD-17, 17-item Hamilton Rating Scale for Depression.

Statistically significant *p* values were shown in bold.

^*^p < 0.05; ^**^p < 0.01.

Furthermore, by univariate and multivariate linear regression analysis, we found that high score of UPDRS III was independently associated with low SPEM gain at 0.1 Hz (multivariate regression: *β* = −0.004, 95% CI: −0.008 to-0.001, *p* = 0.025) and 0.4 Hz (multivariate regression: *β* = −0.006, 95% CI: −0.01 to-0.002, *p* = 0.008) in *de novo* PD. The association between UPDRS III and SPEM gain at 0.2 Hz was only found in univariate linear regression analysis (*β* = −0.003, 95% CI: −0.006 to-0.0004, *p* = 0.023), but not in multivariate linear regression analysis (Table 4).

Oculomotor analysis revealed that saccadic latency was not associated with other oculomotor dynamics such as saccadic accuracy or SPEM gain in PD.

## Discussion

This is the first cross-sectional study in China exploring the oculomotor performances in *de novo*, drug naïve PD patients. We observed that newly diagnosed PD patients had prolonged saccadic latency, poorer saccadic accuracy and lower SPEM gain relative to HCs. Impaired oculomotor performances were found to be significantly associated with PD duration and motor severity. No obvious difference in saccadic and SPEM was found between ET and *de novo* PD patients.

Based on prior anatomical and neuroimaging studies (Hikosaka et al., 2000), it was speculated that oculomotor abnormalities may exist in the early stage of PD, even in the prodromal disease stage. However, there have only been a few studies with conflicting results on whether ocular movements are impaired in *de novo* PD (Bares et al., 2003; Marino et al., 2010; Linder et al., 2012;Antoniades et al., 2015; Hanuška et al., 2019). An overview of the comparisons among the previous studies is shown in Table 5. Different ocular record tools, sampling rates, saccade tasks and sample size may explain the disparity of the results. We found that *de novo* PD had prolonged saccadic latency, which was consistent with Linder’s study (Linder et al., 2012). Also, such kind of patients exhibited lower SPEM gain, as showed in previous two studies (Bares et al., 2003; Marino et al., 2010). However, Linder et al. demonstrated a marginal decrease of SPEM gain in early PD without statistical significance (Linder et al., 2012). The potential reason may be that some patients were under dopaminergic medication in Linder’s study. Unlike the current study, Antoniades et al. (2015) and Hanuška et al. (2019) found no discernible difference in saccadic latency between PD and HCs, possibly due to the relatively small sample size. However, these two studies found that anti-saccade appeared to offer a priority over prosaccade in distinguishing early PD from HCs. Further multi-center studies using standardized ocular record tools and tasks will be conducted to validate oculomotor performance in *de novo* PD.

**Table 5:** Serial studies on eye movement evaluations in *de novo* PD patients.

Study	Sample size, PD/HC	PD treatment	Age and disease duration of PD (mean, years)	VNG machine	Sampling rate	Oculomotor task	Oculomotor characteristics		Clinical associated factors with oculomotor abnormality in PD
							Saccade	SPEM gain	
Bares et al. (2003), Czech Republic	21/21	Drug naïve	59/2.2	Multichannel Brain-Quick EEG/EMG device (Micromed Ltd., Mogliano, Italy)	/	SPEM	/	Reduced SPEM gain in PD	/
Marino et al. (2010), Italy	10/10	Drug naïve	58.5/NA	Vision-based non-intrusive eye tracker	240 Hz	SPEM	/	Reduced pursuit ocular movements in PD	None
Linder et al. (2012), Sweden	105/38	Drug naïve or under dopaminergic medication within 3 months	70/1.2	VNG Ulmer, Synapsys, Marseille, France	/	Prosaccade & SPEM	Longer saccadic latency in PD	No significant difference	Total axial motor scores were associated with saccadic velocity, precision and SPEM gain
Antoniades et al. (2015), UK	19/20	Drug naïve	68/0.67	Head-mounted oculometer	/	Prosaccade & anti-saccade	No difference in prosaccadic latency, but higher AERs in PD	/	AERs were associated with motor severity
Hanuška et al. (2019), Czech Republic	18/25	Drug naïve	62.6/1.6	Binocular video-based eye tracker (mobile eBT, Eyebrain, Ivry-sur-Seine, France)	300 Hz	Prosaccade & anti-saccade	No difference in saccadic latency. Higher AERs and lower horizontal prosaccadic gain in PD	/	/
The current study (2022), China	75/46	Drug naïve	64.5/1.0	Visual Eyes 4 channel VNG (Micromedical Technologies, USA)	120 Hz	Prosaccade & SPEM	Longer saccadic latency in PD	Lower SPEM gain in PD	Prosaccadic latency was associated with disease duration, whereas SPEM gain was associated with motor severity

HC, healthy control; PD, Parkinson’s disease; VNG, videonystagmography; SPEM, smooth pursuit eye movements; AERs, anti-saccadic error rates; NA, not available.

Furthermore, we found that saccadic latency was associated with disease duration and SPEM gain correlated with the motor severity quantified by UPDRS III score in drug naïve PD patients. These findings are consistent with previous studies with patients examined in more advanced stage of the disease (Marino et al., 2007; Terao et al., 2011; Zhang et al., 2018). Neuroimaging evidence has demonstrated that the volume in frontal–parietal regions was reduced and frontal cortex-basal ganglia circuit activity was decreased with disease progression, leading to changes in saccadic parameters (Gorges et al., 2016; Vintonyak et al., 2017; Chen et al., 2022). Anatomical studies have also shown that the basal ganglia may be involved in efficient and automatic SPEM performance(Yoshida and Tanaka, 2009). Neurophysiological recordings in monkeys showed that a subset of neurons in both the external and the internal segments of the globus pallidus modulate SPEM activity (Yoshida and Tanaka, 2009). Significant activation of the caudate nucleus was observed during SPEM in previous imaging studies(O'Driscoll et al., 2000). The SPEM impairments in the current study were in line with the progression of PD motor signs, which may be due to the progressive changes within the basal ganglia. Our results therefore confirmed that the basal ganglia changes in PD patients could impact the operation of cortical and subcortical areas, even at the early stage of the disease. Thus, careful evaluation of the ocular movement performance may provide valuable information for monitoring of the disease.

Similar to previous studies (Gitchel et al., 2013; Wójcik-Pędziwiatr et al., 2016), we found that SPEM in ET patients were abnormal with reduced gain at 0.4 Hz. However, there is much controversy over whether ET patients have eye movement abnormalities in saccadic activity. Our findings are consistent with some previous studies indicating that ET subjects had longer saccadic latency and lower saccadic accuracy than HCs (Gitchel et al., 2013; Wójcik-Pędziwiatr et al., 2017). Two other studies (Helmchen et al., 2003; Trillenberg et al., 2006), however, reported no significant difference in latency of VGSs between the ET and the HC groups. The difference could be explained by the fact that these negative studies only included saccades with a small amplitude (assessed only 10° and 20° saccades). Furthermore, Visser et al. discovered that mean latency in VGSs and anti-saccades performances differed between ET and PD (Visser et al., 2019), which contradicted our findings. It is possible that this is because our PD patients were at an early stage of the disease, and anti-saccade or more advanced tasks were not further investigated as in Visser’s study. As anti-saccade task imposes a higher demand on both cognitive and motor aspects of oculomotor control, future validation studies are needed to investigate the differential value of anti-saccade in ET and *de novo* PD patients.

Although we discovered three ocular parameters with significant difference in separating PD/ET from HCs, the AUC was less than 0.8 in either single or combined parameters (Supplementary Tables1, 2). Furthermore, the ocular parameters could not distinguish ET from *de novo* PD patients. All of these factors made the oculomotor test ineffective as an independent diagnostic tool for the early detection of PD. However, because oculomotor evaluation is a simple, objective, and inexpensive test, it was worth investigating whether combining eye movement evaluation with other clinical features (such as substantia nigra hyperechogenicity) could improve diagnostic of early PD.

The current study has a few limitations that should be mentioned. For starters, because this is a cross-sectional study, only an association between oculomotor parameters and clinical phenotype was discovered. Longitudinal follow-up studies are needed in the future to confirm the importance of ocular movement in the progression of PD. Second, we only have the MMSE for cognitive evaluation in HCs, not the MoCA-BC. Future research should look into how executive dysfunction affects oculomotor performance. Third, in the current study, we used reflexive VGS rather than volitional saccade. Advanced oculomotor tasks (anti-saccade task, for example) in conjunction with fMRI imaging may provide additional clinical value and insight into the underlying neural basis of PD oculomotor impairments. Fourth, we recruited newly diagnosed, unmedicated PD patients; additional follow-up is required to ensure clinical diagnostic accuracy. Post-mortem studies revealed an 80% accuracy in the initial clinical diagnosis of PD disease (Rizzo et al., 2016). When attempting to correlate an additional diagnostic tool with the disease, this may introduce an additional error.

In conclusion, our Chinese population study confirmed that *de novo* PD patients had oculomotor impairments compared to HCs, primarily with prolonged saccadic latency and decreased SPEM gain. Although these eye movement abnormalities in unmedicated PD could not be distinguished from ET, they were related to disease duration and motor severity in *de novo* PD, implying that these parameters could be used to predict disease progression. In the future, longitudinal follow-up studies with detailed phenotypic evaluation, advanced eye movement tasks, and fMRI imaging will be required.

## Data availability statement

The raw data supporting the conclusions of this article will be made available by the authors, without undue reservation.

## Ethics statement

The studies involving human participants were reviewed and approved by the Ethics Committee of Shanghai Ninth People’s Hospital, Shanghai Jiao Tong University School of Medicine. The patients/participants provided their written informed consent to participate in this study.

## Author contributions

M-XZ and QW: conceptualization and formal analysis. M-XZ and WC: methodology and investigation. M-XZ: software and writing—original draft preparation. M-XZ, WC, and QX: validation. M-XZ, QX, YL, LW, and WC: resources. M-XZ, YL, and Y-HJ: data curation. M-XZ, QW, YL, LW, and WC: writing—review and editing. M-XZ and QH: visualization. LZ, Y-RD, J-RL, and WC: supervision. WC: project administration. Y-JC, WC, and J-RL: funding acquisition.All authors contributed to the article and approved the submitted version.

## Funding

This research was funded by 200 talent project from Shanghai Municipal Education Commission-Gaofeng Clinical Medicine Grant Support (20161422), Natural Science Foundation Project from the Shanghai Municipal Science and Technology Commission (22ZR1436900), Shanghai medical guidance program (17411964000), Clinical Research Program of Shanghai Ninth People’s Hospital affiliated to Shanghai Jiao Tong University School of Medicine (JYLJ202003), Project of Biobank from Shanghai Ninth People’s Hospital, Shanghai Jiao Tong University School of Medicine (YBKB202120), and Fundamental Research Program Funding of Shanghai Ninth People’s Hospital affiliated to Shanghai Jiao Tong University School of Medicine (JYZZ155).

## Conflict of interest

The authors declare that the research was conducted in the absence of any commercial or financial relationships that could be construed as a potential conflict of interest.

## Publisher’s note

All claims expressed in this article are solely those of the authors and do not necessarily represent those of their affiliated organizations, or those of the publisher, the editors and the reviewers. Any product that may be evaluated in this article, or claim that may be made by its manufacturer, is not guaranteed or endorsed by the publisher.
