# Supplementary material for: Oculomotor impairments in de novo Parkinson’s disease
Source: Front Aging Neurosci. 2022 Nov 9;14:985679. doi: 10.3389/fnagi.2022.985679 (PMC9682176; doi:10.3389/fnagi.2022.985679)
Supplement: Supplementary file 1 [file Data_Sheet_1.docx]

**Oculomotor** **impairments in *de novo* Parkinson’s disease**

**Supplementary Table 1.**

**Cut-off scores, AUCs, sensitivities and specificities of eye movement parameters between PD and HC.**

| Factor | Cut-off | AUC | *p*-value | Sensitivity (%) | Specificity (%) |
| --- | --- | --- | --- | --- | --- |
| Saccade latency, ms | 205.75 | 0.641 | **0.01^*^** | 80.4 | 50.7 |
| Saccade accuracy, % | 87.75 | 0.681 | **0.001^**^** | 87.0 | 41.3 |
| SPEM gain 0.1Hz | 0.72 | 0.616 | **0.032^*^** | 63.0 | 58.7 |
| SPEM gain 0.2Hz | 0.78 | 0.652 | **0.005^**^** | 65.2 | 64.0 |
| SPEM gain 0.4Hz | 0.78 | 0.66 | **0.003^**^** | 58.7 | 74.7 |
| ^a^ Combined eye movement parameter | 0.61 | 0.78 | **<0.001^***^** | 80.4 | 73.3 |

Notes: PD, Parkinson’s disease; HC, healthy control; AUC, area under the curve; ^a^ Model was obtained by fitting saccade latency, saccade accuracy and SPEM gain 0.4Hz. Statistically significant P values were shown in bold. **p* < 0.05; ***p* < 0.01; and ****p* < 0.001.

**Supplementary Table 2.**

**Cut-off scores, AUCs, sensitivities and specificities of eye movement parameters between ET and HC.**

| Factor | Cut-off | AUC | *p*-value | Sensitivity (%) | Specificity (%) |
| --- | --- | --- | --- | --- | --- |
| Saccade latency, ms | 202.00 | 0.653 | **0.005^**^** | 76.1 | 60.0 |
| Saccade accuracy, % | 87.25 | 0.647 | **0.007^**^** | 89.1 | 63.5 |
| SPEM gain 0.1Hz | 0.69 | 0.586 | 0.114 | 71.7 | 44.6 |
| SPEM gain 0.2Hz | 0.79 | 0.592 | 0.090 | 63.0 | 56.8 |
| SPEM gain 0.4Hz | 0.62 | 0.635 | **0.013^*^** | 80.4 | 40.5 |
| ^a^ Combined eye movement parameter | 0.58 | 0.72 | **<0.001^***^** | 73.9 | 67.6 |

Notes: ET, essential tremor; HC, healthy control; AUC, area under the curve; ^a^ Model was obtained by fitting saccade latency, saccade accuracy and SPEM gain 0.4Hz. Statistically significant P values were shown in bold. ^*^*p* < 0.05; ^**^*p* < 0.01; ^***^*p* < 0.001.
